# Supplementary material for: Genome-Wide Meta-Analysis Identifies Variants in DSCAM and PDLIM3 That Correlate with Efficacy Outcomes in Metastatic Renal Cell Carcinoma Patients Treated with Sunitinib
Source: Cancers (Basel). 2022 Jun 8;14(12):2838. doi: 10.3390/cancers14122838 (PMC9220885; doi:10.3390/cancers14122838)
Supplement: Supplementary file 1 [file cancers-14-02838-s001.zip › Supplementary Figures and Tables.pdf]

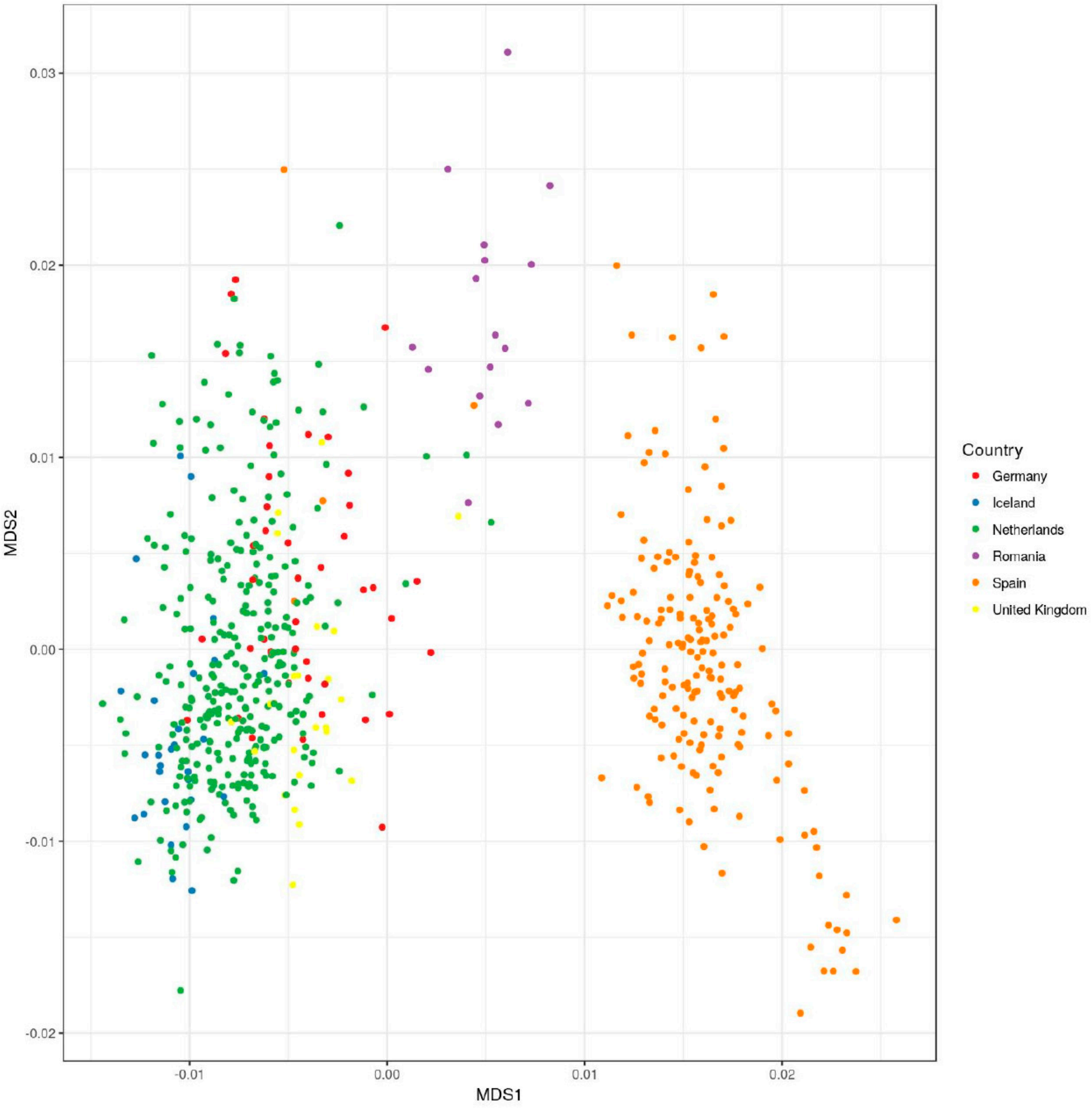
0.03
0.02
0.01
0.00
-0.01
-0.02
MDS2
-0.01
0.00
0.01
0.02
MDS1
Country
Germany
Iceland
Netherlands
Romania
Spain
United Kingdom

Figure S1A

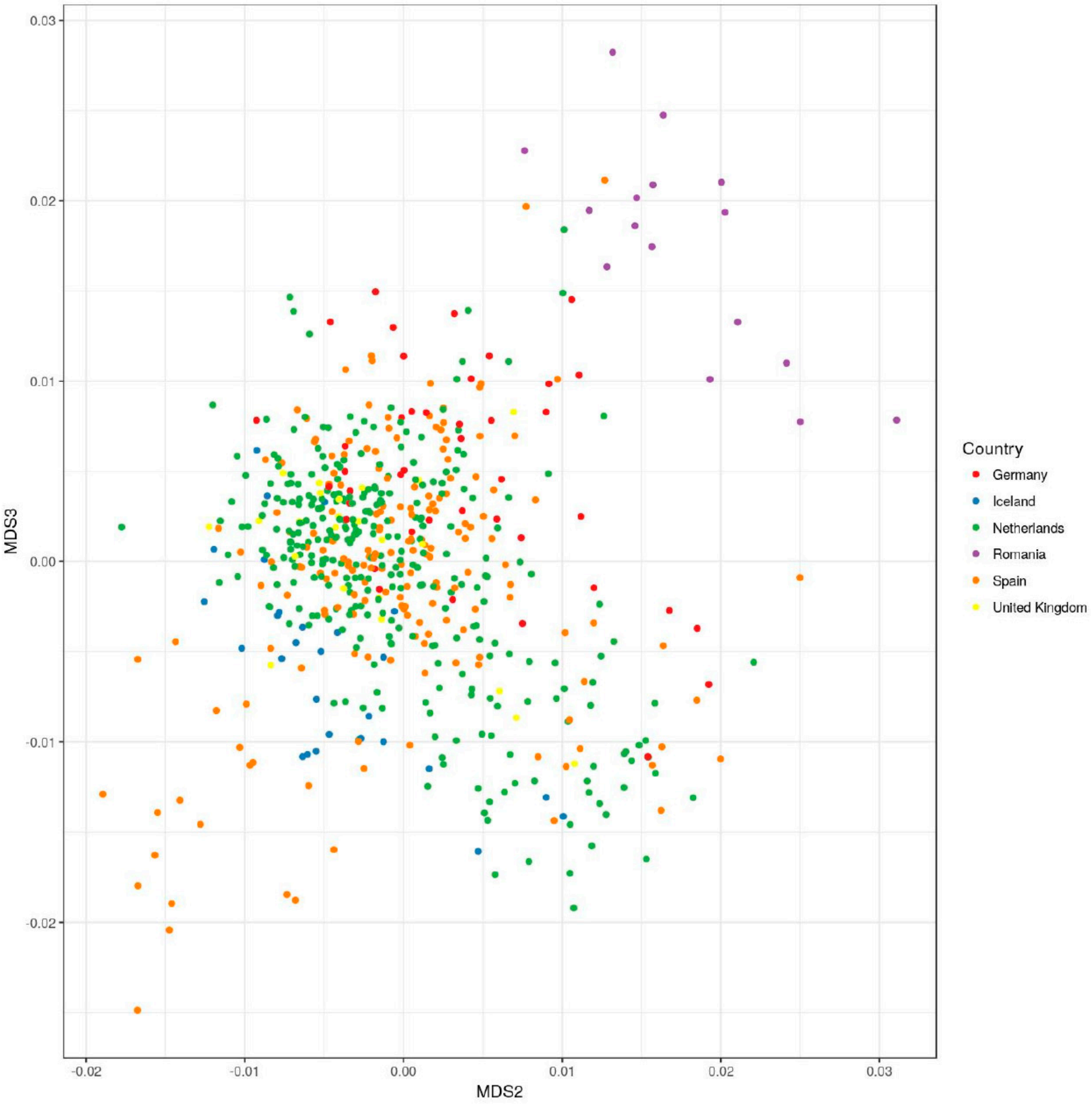

Figure S1B

Figure S1. Multi-dimensional scaling (MDS). The EuroTARGET series was subjected to a MDS analysis in order to detect possible population stratification. MDS computes a low-dimensional map in which distances between original samples are optimally preserved. The distances between individuals is based on IBS dissimilarity. Seven dimensions were used to construct MDS coordinates. The following plots show adjacent coordinates up to the third coordinate. Two main clusters of patients are detectable, with a cluster containing subjects from Spanish centres (SOGUG) and another cluster containing subjects from the other countries (Germany, Iceland, Netherlands, United Kingdom). The Romanian patients were in between these clusters. In addition, MDS showed that 20 individuals were recognized as outliers and were removed from analyses. The information of country of origin corresponds to these MDS component.

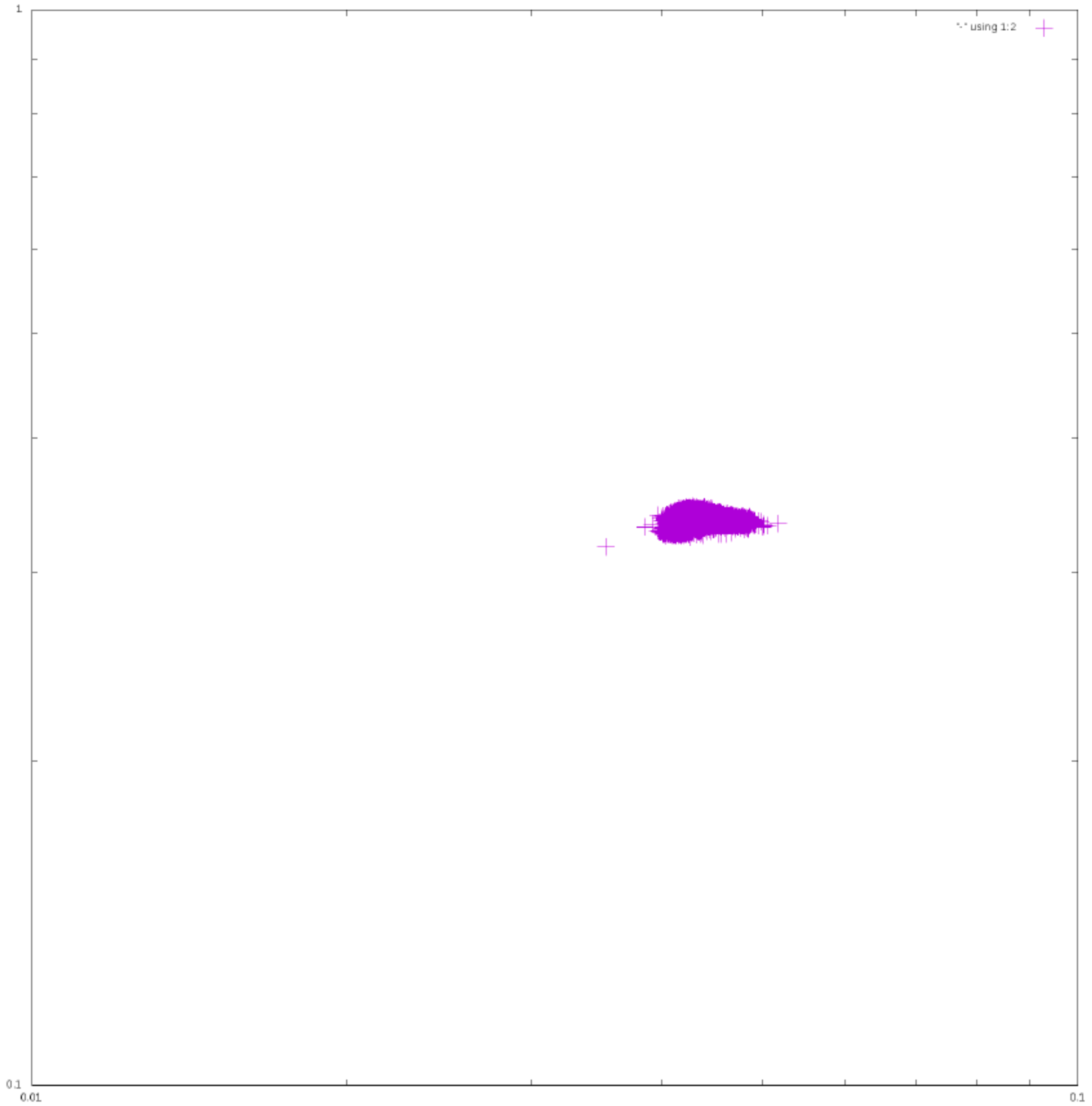

Figure S2. Pairwise IBS values form the basis for MDS and the IBD/IBS clustering. Each point represents a pair of individuals. Frequencies of IBS0, IBS1 are used as coordinates in Figure 2. Based on the plot, no familial clustering was detected.

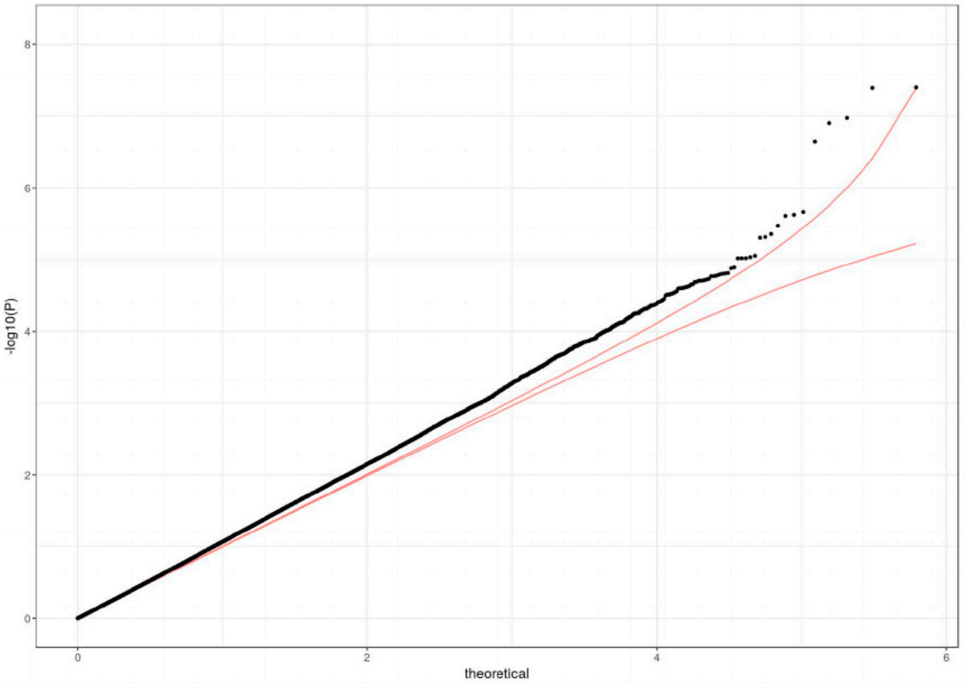

Figure S3a

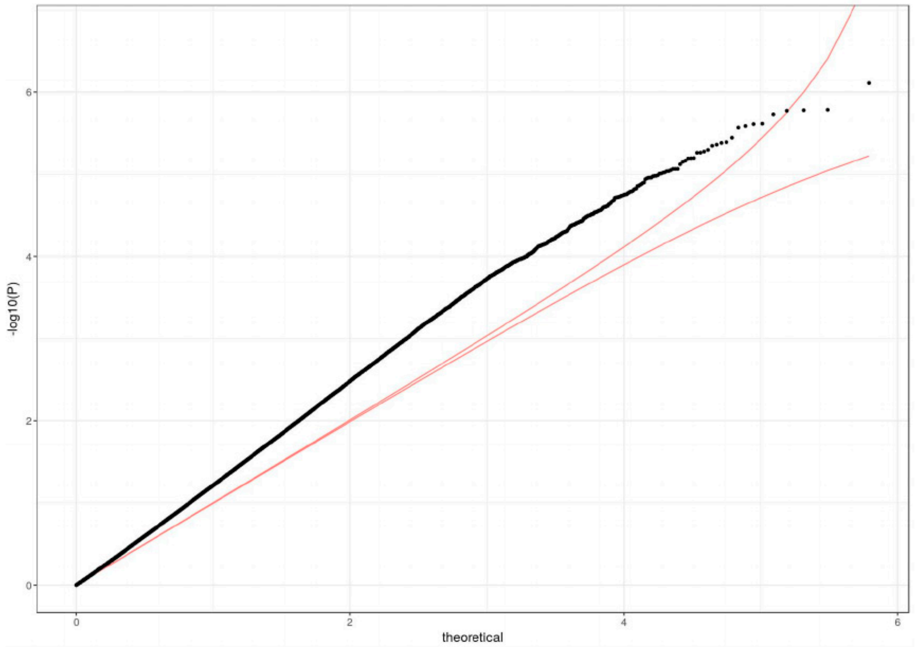

Figure S3b

Figure S3. Quantile-Quantile (QQ) plots for the EuroTARGET cohort. Post association QC was performed by visual inspection of p-values in the Quantile-Quantile (QQ) plots and computation of the inflation factor given as follows: $\lambda = (\text{median}(T_1, \ldots, T_n)/0.675)^2$, where $T_1, \ldots, T_n$ are square roots of χ^2 quantiles. The red lines represent the pointwise 95% confidence intervals. (**a**). Quantile-Quantile (QQ)-plot for PFS analysis in the EuroTARGET cohort. This QQ-plot shows the extent to which the observed distribution of the test statistic

follows the theoretical null distribution. An inflation factor of λ=1.08 was observed. (**b**). Quantile-Quantile (QQ)-plot for OS analysis in the EuroTARGET cohort. This QQ-plot shows the extent to which the observed distribution of the test statistic follows the theoretical null distribution. An inflation factor of λ=1.05 was observed.

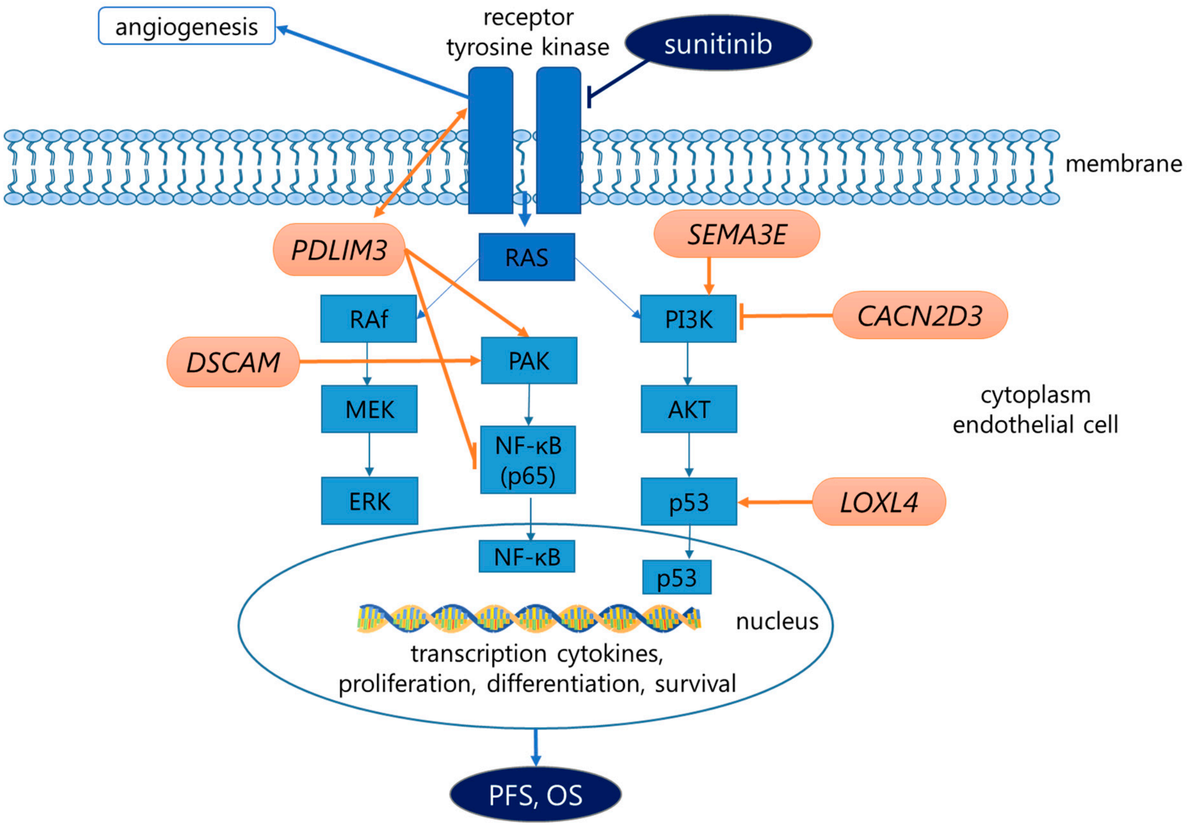

Figure S4. Genetic variants that possibly affect PFS and OS of sunitinib in mRCC. This figure presents novel identified germline DNA variants in genes (presented in orange) that are possibly involved in p21-associated kinases (PAKs) activity and the PI3K/AKT signalling pathway. It is hypothesized that variants in *PDLIM3* and *DSCAM* might modify PAKs activity and influence the PI3K/AKT signalling pathway, and, thereby, modify drug resistance and decrease sunitinib efficacy through NF-κB/IL-6 activation [29-37]. Expression of *CACNA2D3* could block the PI3K/AKT signalling pathways, and LOXL4 expression was related to an improved OS in liver cancer patients with wild-type p53 tumors [36]. SNPs in the *SEMA3E* gene would be essential for gonadotropin-releasing hormone neuron development and acts via PI3K signalling.

Abbreviations: *PDLIM3;* encodes the PDLIM3 protein from the LIM family and contains a PDZ and LIM domain, *DSCAM;* encodes the Down Syndrome Cell Adhesion Molecule (DSCAM), *CACNA2D3*; encodes a member of the alpha-2/delta subunit family, a protein in the voltage-dependent calcium channel complex, *LOXL4*; encodes a member of the lysyl oxidase gene family, *SEMA3E*; encodes semaphoring 3 that belongs to a large family of conserved secreted and membrane associated proteins which possess a semaphorin (Sema) domain and a PSI domain [29-37].

Table S1. List of TKIs or TKI-like antitumor treatments. Patients who received any of these treatments prior to start with sunitinib were excluded from analyses.

**TKI or TKI-like antitumor treatment**
pazopanib + everolimus alternately (ROPETAR study)
sorafenib + capecitabine
axitinib
sunitinib + everolimus (SUNRISES study)
Tivozanib (KRN951)
AZD2171 (cediranib)
Sugen = SU014813 (sunitinib phase I study)
bevacizumab + pazopanib (VDF111687 study)
sorafenib + IL-2
AZD2171 (cediranib) + gevitinib
cabozantinib
cediranib and saractanib/placebo
AURA (=pha-739358) Hierna Bay 57-9352 (phase 1 study)
axitinib + everolimus
vandetanib + selumetinib
Dovitinib (TKI 258)
Everolimus + Cyclofosfamide
Pazopanib + docetaxel

Table S2: Summary of exclusions of markers for QC steps.
At start of the analysis, a set of 680,729 genetic markers was available. After subjecting these markers to QC, 1,405 of the 680,729 SNPs (0.2%) were excluded because of a deviation from HWE, or a MAF <0.01 (N=1,394; 0.2%). All SNP call rates were >97%, and therefore no markers were excluded because of missing genotypes. This resulted in a number of 679,324 genetic markers that meet the quality criteria for statistical analyses.

	Ne	perc	Ncum	missing	HWE	MAF
Missing	0	0.0%	0	0	0	0
HWE	11	0.0%	11	0	11	0
MAF	1394	5.7%	1405	0	0	1394

Abbreviations: *Ne*: number of exclusions; *perc*: percentage of exclusions from total sample after accounting for external exclusions; *Ncum*: cumulative number of excluded markers.

Table S3a,b

Table S3a. GWAS significantly or suggestively associated SNPs with PFS from the EuroTARGET cohort

**SNP**	**Chromosome**	**position**	**gene**	**MAF (%)**	**Allele ***	**P-value**	**Hazard Ratio (HR)**	**beta**	**se**	**Imputation quality**	**Association**
rs28520013	4	186442067	*PDLIM3*	2.30	G/T	4.02E-10	7.26	1.98	0.32	0.530	GWAS significant
rs8126659	21	41682972	*DSCAM*	6.20	C/T	3.27E-09	1.61	0.48	0.75	0.978	
rs1554923	21	41682817	*DSCAM*	6.20	C/A	3.51E-09	1.57	0.45	0.76	0.987	
rs2205096	21	41683405	*DSCAM*	6.30	T/A	5.6E-09	2.50	0.92	0.16	0.936	
rs2205095	21	41676786	*DSCAM*	6.30	T/A	7.76E-09	1.57	0.45	0.76	0.977	
rs7278717	21	41674286	*DSCAM*	6.60	G/C	1.26E-08	1.55	0.44	0.73	0.994	
rs714983	21	41674094	*DSCAM*	6.60	T/G	1.47E-08	1.55	0.44	0.73	0.996	
rs2837567	21	41674814	*DSCAM*	6.20	C/T	2.04E-08	1.59	0.46	0.73	0.984	
rs714982	21	41674108	*DSCAM*	6.70	T/G	2.95E-08	1.55	0.44	0.71	1.000	
rs2837569	21	41680306	*DSCAM*	6.10	G/T	4.04E-08	1.55	0.44	0.72	1.000	
rs111356738	21	41677845	*DSCAM*	5.30	G/A	4.77E-08	2.50	0.92	0.17	0.925	

*The first mentioned alleles are the reference alleles, the second mentioned alleles are the variant alleles.

Table S3b. GWAS significantly or suggestively associated SNPs with PFS from the RIKEN cohort

SNP	Chromosome	position	gene	MAF (%)	Allele*	P-value	Hazard Ratio (HR)	beta	se	Association
rs73158437	22	27769389	*CTA-929C8.8*	13.30	G/A	3.38E-08	1.20	0.19	0.10	GWAS significant
rs73158434	22	27769191	*CTA-929C8.8*	13.30	C/T	3.91E-08	1.20	0.19	0.10	
rs141415598	2	116737488	Position close to *DPP10*	3.90	A/C	6.57E-08	> 100**			GWAS suggestive
rs76222746	2	116751751		3.90	A/G	6.57E-08	> 100***			
rs10198910	2	116718944		3.90	G/A	6.95E-08	> 100****			
rs58368668	4	156183712	Position close to *MAP9*, *GUCY1A3*, *GUCY1B3*, *ASIC5*, *TDO2*, *CTSO*, and *PDGFC*	15.80	T/A	1.82E-07	1.02	0.02	0.10	
rs75209750	4	156184140		15.80	A/T	1.82E-07	1.02	0.02	0.10	
rs12098459	10	19408659	*MALRD1*	4.60	A/G	2.35E-07	1.00	0.001	0.16	
rs34013078	10	19408936	*MALRD1*	4.50	G/T	2.35E-07	0.94	-0.06	0.17	
rs17032673	4	156148386	Position close to *MAP9*, *GUCY1A3*, *GUCY1B3*, *ASIC5*, *TDO2*, *CTSO*, and *PDGFC*	18.00	G/A	2.46E-07	0.92	-0.08	0.09	

*The first mentioned alleles are the reference alleles, the second mentioned alleles are the variant alleles.

**log(HR) = 20.0, ***log(HR) = 14.9, ****log(HR) = 14.8
