# Supplementary material for: Genome-Wide Meta-Analysis Identifies Variants in DSCAM and PDLIM3 That Correlate with Efficacy Outcomes in Metastatic Renal Cell Carcinoma Patients Treated with Sunitinib
Source: Cancers (Basel). 2022 Jun 8;14(12):2838. doi: 10.3390/cancers14122838 (PMC9220885; doi:10.3390/cancers14122838)
Supplement: Supplementary file 1 [file cancers-14-02838-s001.zip › Supplementary File S1a.pdf]

## Eligibility criteria

### Eligibility criteria

In case a certain date is unknown, please use the following guidelines:
- In case the month and year are known: 01-mm-yyyy
- In case only the year is known: 01-01-yyyy
- In case the total date is unknown: uk

| | |
|---|---|
| Metastasis | O Yes<br>O No (In case of "No" a patient can NOT be enrolled in EuroTARGET) |
| Date of diagnosis of metastasis | __ __ -- __ __ -- __ __ __ __ |
| Institute of diagnosis | ..........................................................................................<br>(For the Netherlands: according to NKR codes for hospitals) |
| Date, the informed consent has been signed by the patient | __ __ -- __ __ -- __ __ __ __ |

## General information at diagnosis

### Height

| | |
|---|---|
| Patient's height | ........................................ (in cm) |

### Bilateral renal cell carcinoma

A patient has bilateral renal cell carcinoma, in case a second tumor in the other kidney was detected within three months. If the patient has bilateral renal cell carcinoma, it is not necessary to fill in the information for both individual tumors.

| | |
|---|---|
| Bilateral renal cell carcinoma | O Yes<br>O No<br>O Unknown |
| In case of bilateral renal cell carcinoma:<br>Synchronous occurrence? | O Synchronous (fill in the form using the first tumor)<br>O Non-synchronous (fill in the form using the tumor with the worst prognosis)<br>O Unknown |

### Tumor assessment

Multiple dates should be registered in this form.
In case a certain date is unknown, please use the following guidelines:
- In case the month and year are known: 01-mm-yyyy
- In case only the year is known: 01-01-yyyy
- In case the total date is unknown: uk

This information should be registered only once for each patient at time of recruitment.
In most cases metastasis will be diagnosed together with the primary tumor.
In case metastasis was diagnosed later, please fill in the information below on the PRIMARY TUMOR.

| | |
|---|---|
| Date of initial diagnosis of primary tumor | __ __ -- __ __ -- __ __ __ __ |
| Date of surgery / biopsy for primary tumor | __ __ -- __ __ -- __ __ __ __ |
| Tumor sample on which the pathology diagnosis was based comes from | O Primary tumor<br>O Metastasis<br>O No pathology diagnosis |
| Pathology laboratorium of diagnosis | .........................................................................................<br>(For the Netherlands: according to NKR codes for hospitals) |
| Pathology identification number of specimen | ......................................................................................... |
| Type of renal cell cancer | O Clear renal cell carcinoma<br>O Chromophobe renal cell carcinoma<br>O Collecting duct carcinoma<br>O Papillary renal cell carcinoma<br>O Non-clear cell renal cell carcinoma<br>O Unknown |
| Papillary type | O Papillary type I RCC<br>O Papillary type II RCC<br>O Unknown) |
| Please specify non-clear cell renal cell carcinoma | ............................................................................ |
| | |

| | |
|---|---|
| Presence of sarcomatoid elements | O Yes<br>O No<br>O Unknown |
| Fuhrman grade | O Grade I<br>O Grade II<br>O Grade III<br>O Grade IV<br>O Unknown |
| Presence of tumor necrosis | O Yes<br>O No<br>O Unknown |

## cTNM classification

According to TNM staging system 7 (2009), based on clinical information. For instructions, please see header row.

**TNM staging system - clinical**
Indicate the TNM stage according to the TNM classification 7 (2009) from clinical information. In case a previous edition of the TNM staging system was used, re-evaluate the TNM stage according to this new edition (2009).

**Summary of the changes in the TNM classification 7 (2009)**
T2 has been subdivided in:
T2a - Tumor more than 7 cm but not more than 10 cm
T2b - Tumor more than 10 cm, limited to the kidney
T3 and T4 got changes concerning the text:
T3a - Invasion into the adrenal gland is removed from this stage (now T4)
T4 - Tumor invades beyond Gerota fascia (including contiguous extension into ipsilateral adrenal gland)

| | | | |
|---|---|---|---|
| cT- Primary Tumor | O TX<br>O T0<br>O T1<br>O T1a<br>O T1b | O T2<br>O T2a<br>O T2b | O T3<br>O T3a<br>O T3b<br>O T3c<br>O T4 |
| cN- Regional Lymph Nodes | O NX<br>O N0<br>O N1<br>O N2 | | |
| cM- Distant | O M0<br>O M1 | | |

## pTNM classification

According to TNM staging system 7 (2009), based on pathology. For instructions, please see the header row.

**TNM staging system - pathology**
Indicate the TNM stage according to the TNM classification 7 (2009) from the pathology report. In case a previous edition of the TNM staging system was used, re-evaluate the TNM stage according to this new edition (2009). In case a patient did not get surgery the pTNM cannot be assessed, since no pathological grading took place. In this case, indicate the TNM classification based on clinical information.

**Summary of the changes in the TNM classification 7 (2009)**
T2 has been subdivided in:
T2a - Tumor more than 7 cm but not more than 10 cm
T2b - Tumor more than 10 cm, limited to the kidney
T3 and T4 got changes concerning the text:
T3a - Invasion into the adrenal gland is removed from this stage (now T4)
T4 - Tumor invades beyond Gerota fascia (including contiguous extension into ipsilateral adrenal gland)

| | |
|---|---|
| pT- Primary Tumor | O TX<br>O T0<br>O T1<br>O T1a<br>O T1b<br>O T2<br>O T2a<br>O T2b<br>O T3<br>O T3a<br>O T3b<br>O T3c<br>O T4 |
| pN- Regional Lymph Nodes | O NX<br>O N0<br>O N1<br>O N2 |
| pM- Distant | O M0<br>O M1 |
| | O pTNM classification not done |

## Co-morbidities at start treatment of metastasis

If co-morbidities exist at the time of start of treatment of metastasis, these co-morbidities should be registered here. A co-morbidity is NOT related to the cancer-specific treatment, and NOT related to the disease itself.

| | |
|---|---|
| Other malignancy<br>excl. non-melanoma skin cancer and carcinoma in situ of the cervix uteri | O No<br>O Yes, please specify ……………………………………………………<br>O Unknown |
| Diabetes mellitus | O No<br>O Yes<br>O Unknown |
| Hypertension | O No<br>O Yes<br>O Unknown |
| in case of “yes”, currently treated with medication? | O No<br>O Yes<br>O Unknown |
| Cardiovascular disease<br>e.g. Intermittent claudication, bifurcation prosthesis, percutaneous transluminal angioplasty (PTA), tube prosthesis, abdominal aortic aneurysm, aortic aneurysm, generalized atherosclerosis, peripheral vascular disease, embolectomy, post myocardial infarction, myocardial infarction, angina pectoris, coronary artery bypass graft (CABG), coronary heart disease, percutaneous transluminal coronary angioplasty (PTCA), heart failure, cardiomyopathy, cerebrovascular accident (CVA) in history (not a transient ischemic attack (TIA), cerebral artery disease, cerebral hemorrhage, post carotid endarterectomy, heartvalve prosthesis (aortic valve prosthesis, mitral valve prosthesis), valvular heart disease (mitral, tricuspid, aortic or pulmonary valve stenosis or regurgitation), post heart transplantation, thrombosis, pulmonary embolism, pelvic vein thrombosis, arrhythmias, atrial fibrillation, ventricular fibrillation, AV-block, artificial pacemaker, cardioversion.<br>Although DVT actually falls in this category, we would like to have a separate registration of DVT. | O No<br>O Yes, please specify ……………………………………………………<br>O Unknown |
| Deep vein thrombosis<br>Although DVT actually falls in category "cardiovascular diseases", we would like to have a separate registration of DVT. | O No<br>O Yes, most recent year of diagnosis __ __ -- __ __ -- __ __ __ __<br>O Unknown |
| Other clotting disorder<br>e.g. haemophilia | O No<br>O Yes, please specify ……………………………………………………<br>O Unknown |

| | |
|---|---|
| Lung disease<br>e.g. CARA, emphysema, chronic bronchitis, chronic obstructive pulmonary disease, obstructive pulmonary disease, bronchial hyperreactivity, pulmonary fibrosis, post-lung transplantation. | O No<br>O Yes, please specify ………………………………………………<br>O Unknown |
| Gastro intestinal<br>e.g. peptic ulcer, gastric ulcer, duodenal ulcer, (reflux) esophagitis, post gastrectomy, total or partial (B1, B2-resection), inflammatory bowel disease (Morbis Crohn, CU (Colitis Ulcerosa) (no familial adenomatosis polyposis), liver disease (e.g. cirrhosis, hepatitis A, B, C, non-A and non-B), post liver transplantation, diverticulitis. | O No<br>O Yes, please specify ………………………………………………<br>O Unknown |
| Urinary tract and genital organs<br>e.g.chron. glomerulonephritis (CGN), chron. pyelonephritis (CPN), chronic renal failure, nephrotic syndrome, post kidney transplantation, dialysis (haemodialysis, CIPD, peritoneal dialysis). | O No<br>O Yes, please specify ………………………………………………<br>O Unknown |
| Muscles, joints and connective tissue<br>e.g. connective tissue diseases (e.g., Sarcoidosis = M. Besnier Boeck, Wegener, periarteriitis nodosa (PAN), Systemic lupus erythematosus (SLE), rheumatoid arthritis (RA) | O No<br>O Yes, please specify ………………………………………………<br>O Unknown |
| Central and peripheral NS<br>e.g. dementia, Alzheimer's disease, dementia syndrome, hemiplegic, hemiparesis, quadriplegic, paraplegic, psychiatric disorders (this refers to severe chronic psychiatric problems, e.g. hospitalized in a psychiatric hospital or ward, severe depression, psychosis or schizophrenia), Parkinson's disease. | O No<br>O Yes, please specify ………………………………………………<br>O Unknown |
| Metabolic disease<br>e.g. haemophilia, hypo-/hyperthyreoidia | O No<br>O Yes, please specify ………………………………………………<br>O Unknown |
| Infectious disease<br>Only chronic infectious diseases, e.g. HIV-positive, AIDS, tuberculosis. No acute infectious diseases, e.g. pneumonia. | O No<br>O Yes, please specify ………………………………………………<br>O Unknown |
| Congenital disorder<br>e.g. Down syndrome, clubfoot, facial clefts, 18q syndrome, sickle cell disease | O No<br>O Yes, please specify ………………………………………………<br>O Unknown |
| Other disorder | O No<br>O Yes, please specify ………………………………………………<br>O Unknown |

## Available patient material

### Blood sample

In case a certain date is unknown, please use the following guidelines:
- In case the month and year are known: 01-mm-yyyy
- In case only the year is known: 01-01-yyyy
- In case the total date is unknown: uk

| | |
|---|---|
| Blood sample | O Yes<br>date of venepuncture __ __ -- __ __ -- __ __ __ __<br>O No |

### Frozen tumor sample

| | |
|---|---|
| Frozen tumor sample available for EuroTARGET | O Yes<br>O No |
| Available tumor material | O Primary tumor<br>O Metastasis<br>O Primary tumor and metastasis |

### Paraffin-embedded tumor sample

| | |
|---|---|
| Paraffin-embedded tumor sample available for EuroTARGET | O Yes<br>O No |
| Available tumor material | O Primary tumor<br>O Metastasis<br>O Primary tumor and metastasis |

## Treatment line

### Treatment of metastasis

Information collected during treatment regimes for metastasis.
When another drug is administered a new treatment line starts.
Please use one form per treatment line!
"1st line treatment of metastasis" should always be filled in. 2nd, 3rd, 4th and 5th treatment lines should only be filled in in case of (multiple) medication change(s).

Multiple dates should be registered in this form. In case a certain date is unknown, please use the following guidelines:
- In case the month and year are known: 01-mm-yyyy
- In case only the year is known: 01-01-yyyy
- In case the total date is unknown: uk

| | |
|---|---|
| Treatment line no | O 1 or palliative care or best supportive care<br>O 2<br>O 3<br>O 4<br>O 5 |

### Clinical data at start of treatment of metastasis

| | |
|---|---|
| In which hospital is this treatment line administered | ………………………………………………………………………………<br>(For the Netherlands: according to NKR codes for hospitals) |
| Weight of the patient | …………………………………… (in kg) |
| WHO performance status<br>Register the WHO performance status. Copy exactly what is written in the medical record. Do not interpret the performance status yourself. When the WHO performance status cannot be found, but the Karnofsky score is available, the WHO performance status can be derived from the Karnofsky score. | O 0. Fully active, able to carry on all predisease activities without restrictions<br>O 1. Restricted in physically strenuous activity but ambulatory and able to carry out work of a light or sedentary nature<br>O 2. Ambulatory and capable of all self care but unable to carry out any \ work activities. Up and about more than 50 percent of waking hours<br>O 3. Capable of only limited self-care, confined to bed or chair for 50 percent or more of waking hours<br>O 4. Completely disabled. Cannot carry on any self-care. Totally confined to bed or chair<br>O Unknown |

| *WHO* | *Karnofsky score* | *Description* |
|---|---|---|
| 0 | 100 | Normal no complaints, no evidence of disease. |
| 0 | 90 | Able to carry on normal activity; minor signs or symptoms of disease. |
| 1 | 80 | Normal activity with effort; some signs or symptoms of disease. |
| 1 | 70 | Cares for self; unable to carry on normal activity or to do active work. |
| 2 | 60 | Requires occasional assistance, but is able to care for most of his personal needs. |
| 2 | 50 | Requires considerable assistance and frequent medical care. |
| 3 | 40 | Disabled; requires special care and assistance. |
| 3 | 30 | Severely disabled; hospital admission is indicated although death not imminent. |
| 4 | 20 | Very sick; hospital admission necessary; active supportive treatment necessary. |
| 4 | 10 | Moribund; fatal processes progressing rapidly. |
| 4 | 0 | Dead |

## Number of locations with metastases

| | |
|---|---|
| Number of locations with metastases<br>Indicate the number of locations (number of sites) with metastases at the start of this treatment line. Also include lymph node metastases. Regional and distant lymph node metastases count as one site. | O 1<br>O 2<br>O 3<br>O 4<br>O > 4 |

## Location of metastases

| | |
|---|---|
| Location of metastases | O Lung<br>O Lymph nodes (regional and/or distant)<br>O Liver<br>O Bone<br>O Brain<br>O Adrenal gland<br>O Other, please specify ............................................................. |

## Lab values at start of treatment of metastasis

Enter the lab values at the start of this treatment line. Values must not be older than 3 months before start treatment. When necessary, the characters < or > can be used in the fields below.

| | | | |
|---|---|---|---|
| Hemoglobin | ................................ | O mmol/L<br>O g/dl | (calculated to 1 decimal place) |
| Leukocytes | ................................ | x $10^9$/L (=$10^3$/µl) | (calculated to 1 decimal place) |
| Percentage of neutrophils | ................................ | % | (calculated to 1 decimal place) |
| Number of neutrophils | ................................ | x $10^9$/L | (calculated to 2 decimal places) |
| Trombocytes (platelets) | ................................ | x $10^9$/L (=$10^3$/µl) | |
| Creatinine | ................................ | O µmol/L<br>O mg/dl | (calculated to 1 decimal place) |
| Albumin | ................................ | g/L | |
| Calcium | ................................ | O mmol/L | (calculated to 2 decimal places) |
| Total bilirubin | ................................ | O µmol/L<br>O mg/dl | (calculated to 1 decimal place) |
| Alkaline phosphatase | ................................ | U/L | |
| Aspartate aminotransferase (SGOT/ AST/ ASAT) | ................................ | U/L | |
| Alanine transaminase (SGPT/GUID/ALT/ALAT) | ................................ | U/L | |
| LDH | ................................ | units/L | |

## Drug treatment

| | |
|---|---|
| Which drug(s) is/are being used | O None, palliative or best supportive care<br>O Interferon-alpha (IFN-a)<br>O Interferon-alpha (IFN-a) + vinblastine<br>O Interleukin 2<br>O Sunitinib<br>O Sorafenib<br>O Bevacizumab + Interferon-alpha (IFN-a)<br>O Temsirolimus<br>O Everolimus<br>O Pazopanib<br>O Other, please specify ..............................................................<br>O Unknown |

## Dosing schemes

Please specify dose, frequency etc. in detail below.

A treatment line can consist of different cycles. This is the case for the treatment with Sunitinib. Usually Sunitinib is given during four weeks (weeks on), followed by a two-week rest-period (weeks off). This six-week period is equal to one cycle.

One dosing scheme is a (number of ) treatment cycle(s). In case the treatment cycle has been changed (e.g. in case of dose reduction, change in frequency etc.), please fill in the new treatment cycle in dosing scheme 2, 3, etc. You can add extra schemes by clicking on the + button.

A treatment line can also consist of a treatment given during a certain number of weeks/ months. No cycles are involved. This is the case for the treatment with Interferon-alpha, Sorafenib, Everolimus, Pazopanib, Bevacizumab (+ interferon alpha) and Temsirolimus. Use "n/a" in the "weeks on", "weeks off", "total amount per cycle' and "no of cycles with this scheme" fields.

A treatment may consist of a combination of drugs (e.g. Bevacizumab + Interferon alpha). If this is the case, use two schemes for registering this treatment.

In case a new treatment line has started (change of medication), use a new therapy form ("2nd, 3rd, 4th, 5th line treatment of metastasis") for registering this line.

| 1. | | | | | |
|---|---|---|---|---|---|
| | Start Date 1 | __ __ -- __ __ -- __ __ __ __ | | | |
| | Dose 1 | ……………………………. | O mg<br>O million IU<br>O ml<br>O mmol<br>O other, please specify ……………………………………<br>O unknown | (calculated to 2 decimal places) | |
| | Frequency 1 | O 0.5 x<br>O 1 x<br>O 2 x<br>O 3 x<br>O unknown | per 1 | O day<br>O 3 days<br>O week<br>O month<br>O unknown | |
| | Weeks on treatment 1 | …………… | Weeks off treatment 1 | ……………. | In case no cycles are involved (eg., interferon alpha) use **n/a.** |
| | Total amount per cycle 1 | …………… | No of cycles with this scheme 1 | ……………. | Please use the unit chosen above. In case no cycles are involved (eg., interferon alpha) use **n/a.** |
| | Stop date 1 | __ __ -- __ __ -- __ __ __ __ | Reason for stop/change 1 | O toxicity grade 1 or 2<br>O toxicity grade 3 or 4<br>O progression / relapse<br>O condition of patient<br>O wishes of patiënt<br>O patient has died<br>O other, please specify ……………………………<br>O unknown | |

| 2. | | | | | |
|---|---|---|---|---|---|
| | Start Date 2 | __ __ -- __ __ -- __ __ __ __ | | | |
| | Dose 2 | ……………………………. | O mg<br>O million IU<br>O ml<br>O mmol<br>O other, please specify ……………………………………<br>O unknown | (calculated to 2 decimal places) | |
| | Frequency 2 | O 0.5 x<br>O 1 x<br>O 2 x<br>O 3 x<br>O unknown | per 2 | O day<br>O 3 days<br>O week<br>O month<br>O unknown | |
| | Weeks on treatment 2 | | Weeks off treatment 2 | ……………. | In case no cycles are involved (eg., interferon alpha) use **n/a.** |
| | Total amount per cycle 2 | …………… | No of cycles with this scheme 2 | ……………. | Please use the unit chosen above. In case no cycles are involved (eg., interferon alpha) use **n/a.** |
| | Stop date 2 | __ __ -- __ __ -- __ __ __ __ | Reason for stop/change 2 | O toxicity grade 1 or 2<br>O toxicity grade 3 or 4<br>O progression / relapse<br>O condition of patient<br>O wishes of patiënt<br>O patient has died<br>O other, please specify ……………………………<br>O unknown | |

3.

| | | | | | |
|---|---|---|---|---|---|
| Start Date 3 | __ __ -- __ __ -- __ __ __ __ | | | | |
| Dose 3 | ……………………………… | O mg<br>O million IU<br>O ml<br>O mmol<br>O other, please specify ……………………………………<br>O unknown | (calculated to 2 decimal places) | | |
| Frequency 3 | O 0.5 x<br>O 1 x<br>O 2 x<br>O 3 x<br>O unknown | per 3 | O day<br>O 3 days<br>O week<br>O month<br>O unknown | | |
| Weeks on treatment 3 | | Weeks off treatment 3 | ……………. | In case no cycles are involved (eg., interferon alpha) use **n/a.** | |
| Total amount per cycle 3 | …………… | No of cycles with this scheme 3 | ……………. | Please use the unit chosen above. In case no cycles are involved (eg., interferon alpha) use **n/a.** | |
| Stop date 3 | __ __ -- __ __ -- __ __ __ __ | Reason for stop/change 3 | O toxicity grade 1 or 2<br>O toxicity grade 3 or 4<br>O progression / relapse<br>O condition of patient<br>O wishes of patiënt<br>O patient has died<br>O other, please specify ……………………………<br>O unknown | | |

4.

| | | | | | |
|---|---|---|---|---|---|
| Start Date 4 | __ __ -- __ __ -- __ __ __ __ | | | | |
| Dose 4 | ……………………………… | O mg<br>O million IU<br>O ml<br>O mmol<br>O other, please specify ……………………………………<br>O unknown | (calculated to 2 decimal places) | | |
| Frequency 4 | O 0.5 x<br>O 1 x<br>O 2 x<br>O 3 x<br>O unknown | per 4 | O day<br>O 3 days<br>O week<br>O month<br>O unknown | | |
| Weeks on treatment 4 | | Weeks off treatment 4 | ……………. | In case no cycles are involved (eg., interferon alpha) use **n/a.** | |
| Total amount per cycle 4 | …………… | No of cycles with this scheme 4 | ……………. | Please use the unit chosen above. In case no cycles are involved (eg., interferon alpha) use **n/a.** | |
| Stop date 4 | __ __ -- __ __ -- __ __ __ __ | Reason for stop/change 4 | O toxicity grade 1 or 2<br>O toxicity grade 3 or 4<br>O progression / relapse<br>O condition of patient<br>O wishes of patiënt<br>O patient has died<br>O other, please specify ……………………………<br>O unknown | | |

| (Concomitant) non drug treatment - Surgery | |
|---|---|
| | O No surgery |
| Date of surgery | __ __ -- __ __ -- __ __ __ __ |
| Surgery type | O Open nephrectomy<br>O Laparoscopic nephrectomy<br>O Open partial nephrectomy<br>O Laparoscopic partial nephrectomy<br>O Adrenalectomy<br>O Lymfadenectomy<br>O Thrombectomy<br>O Embolisation<br>O Radio Frequency Ablation (RFA)<br>O Cryosurgery<br>O Metastasectomy<br>O Nephrectomy + metastasectomy (in one session)<br>O Partial nephrectomy + metastasectomy (in one session)<br>O Nephrectomy + other (in one session)<br>O Partial nephrectomy + other (in one session)<br><br>O Other, please specify ..........................................................................<br>O Unknown |

| (Concomitant) non drug treatment - Radiotherapy | |
|---|---|
| | O No radiotherapy |
| Start date | __ __ -- __ __ -- __ __ __ __ |
| Stop date | __ __ -- __ __ -- __ __ __ __ |
| Total dose | ................................ Gy. |
| Site of radiotherapy | .................................................................................... |
| Subjective (symptomatic) response to radiotherapy | O No<br>O Yes<br>O Unknown |

## Toxicities

The items in this table must be updated on an ongoing basis.

Please collect only adverse events which are (likely) CAUSALLY related to the treatment.
All grade 3, 4 and 5 toxicities (NOT grade 1 and 2) developed until one month after the last treatment should be reported. Register per treatment line each toxicity only once, with the highest CTC grade reached. If a toxicity existed before the diagnosis of renal cell carcinoma was made, but the CTC grade has increased after diagnosis, this toxicity must also be reported. In this case the starting date is the day that the toxicity worsens.

If the CTC grade was not specified by the physician in the medical record of the patient, specify the grade of each specific toxicity yourself according to CTCAE version 4.0. The CTC grade refers to the severity of the toxicity. For each specific toxicity, the CTCAE guidelines give a unique clinical description which corresponds to the degree of toxicity. You can find the CTCAE at the following page: http://evs.nci.nih.gov/ftp1/CTCAE/CTCAE_4.03_2010-06-14_QuickReference_8.5x11.pdf. Always consult these guidelines to find the specific CTC grade for the toxicity to be reported.

The following symptoms are not required to report as toxicity:
- Symptoms that can be linked to (progress/relapse of) renal cell carcinoma.
- Symptoms that already existed in the same degree of seriousness at the time of diagnosis.
- Symptoms that can be regarded as symptoms of a previously reported other toxicity (e.g. swallowing difficulties due to mucositis).

Fever must be registered. Please also report whether fever is accompanied by neutropenia or not.
Note: If fever occurs as a result of a (allergic) reaction to the medication, the so-called "drug fever", this fever should also be reported as a toxicity.

| | **Toxicity** If not specified by the physician, consult terminology used in the PDF file mentioned above | **CTC grade** If not specified by the physician, consult the guidelines in the PDF file mentioned above to find the specific CTC grade for the specific toxicity | **Determined by** | **Treatment to which the toxicity is related*** | **Start date of toxicity** | **End date of toxicity** | **Is toxicity treated?** |
|---|---|---|---|---|---|---|---|
| | | **Grade 3**: Serious toxicity: influences general daily life activities, hospitalisation sometimes required<br>**Grade 4**: Life-threatening toxicity: always requires hospitalization<br>**Grade 5**: Death due to toxicity | Indicate whether the CTC grade was specified by the **physician** in the medical record of the patient, or whether the grade was interpreted by the data manager from the medical record (**DM**) | Report the treatment to which the toxicity is (very likely) related. | Note the date the toxicity has started. | Note the date the toxicity has ended. | Is the toxicity treated? |
| 1. | ……………… | ………………… | O physician<br>O DM | ………………… | _ _ - _ _ - _ _ _ _ | _ _ - _ _ - _ _ _ _ | O no<br>O yes |
| 2. | ……………… | ………………… | O physician<br>O DM | ………………… | _ _ - _ _ - _ _ _ _ | _ _ - _ _ - _ _ _ _ | O no<br>O yes |
| 3. | ……………… | ………………… | O physician<br>O DM | ………………… | _ _ - _ _ - _ _ _ _ | _ _ - _ _ - _ _ _ _ | O no<br>O yes |
| 4. | ……………… | ………………… | O physician<br>O DM | ………………… | _ _ - _ _ - _ _ _ _ | _ _ - _ _ - _ _ _ _ | O no<br>O yes |
| 5. | ……………… | ………………… | O physician<br>O DM | ………………… | _ _ - _ _ - _ _ _ _ | _ _ - _ _ - _ _ _ _ | O no<br>O yes |

| | **Toxicity** If not specified by the physician, consult terminology used in the PDF file mentioned above | **CTC grade** If not specified by the physician, consult the guidelines in the PDF file mentioned above to find the specific CTC grade for the specific toxicity | **Determined by** | **Treatment to which the toxicity is related*** | **Start date of toxicity** | **End date of toxicity** | **Is toxicity treated?** |
|---|---|---|---|---|---|---|---|
| 6. | ……………… | ………………… | O physician<br>O DM | ………………… | _ _ - _ _ - _ _ _ _ | _ _ - _ _ - _ _ _ _ | O no<br>O yes |
| 7. | ……………… | ………………… | O physician<br>O DM | ………………… | _ _ - _ _ - _ _ _ _ | _ _ - _ _ - _ _ _ _ | O no<br>O yes |
| 8. | ……………… | ………………… | O physician<br>O DM | ………………… | _ _ - _ _ - _ _ _ _ | _ _ - _ _ - _ _ _ _ | O no<br>O yes |
| 9. | ……………… | ………………… | O physician<br>O DM | ………………… | _ _ - _ _ - _ _ _ _ | _ _ - _ _ - _ _ _ _ | O no<br>O yes |
| 10. | ……………… | ………………… | O physician<br>O DM | ………………… | _ _ - _ _ - _ _ _ _ | _ _ - _ _ - _ _ _ _ | O no<br>O yes |
| 11. | ……………… | ………………… | O physician<br>O DM | ………………… | _ _ - _ _ - _ _ _ _ | _ _ - _ _ - _ _ _ _ | O no<br>O yes |
| 12. | ……………… | ………………… | O physician<br>O DM | ………………… | _ _ - _ _ - _ _ _ _ | _ _ - _ _ - _ _ _ _ | O no<br>O yes |
| 13. | ……………… | ………………… | O physician<br>O DM | ………………… | _ _ - _ _ - _ _ _ _ | _ _ - _ _ - _ _ _ _ | O no<br>O yes |
| 14. | ……………… | ………………… | O physician<br>O DM | ………………… | _ _ - _ _ - _ _ _ _ | _ _ - _ _ - _ _ _ _ | O no<br>O yes |
| 15. | ……………… | ………………… | O physician<br>O DM | ………………… | _ _ - _ _ - _ _ _ _ | _ _ - _ _ - _ _ _ _ | O no<br>O yes |
| 16. | ……………… | ………………… | O physician<br>O DM | ………………… | _ _ - _ _ - _ _ _ _ | _ _ - _ _ - _ _ _ _ | O no<br>O yes |
| 17. | ……………… | ………………… | O physician<br>O DM | ………………… | _ _ - _ _ - _ _ _ _ | _ _ - _ _ - _ _ _ _ | O no<br>O yes |
| 18. | ……………… | ………………… | O physician<br>O DM | ………………… | _ _ - _ _ - _ _ _ _ | _ _ - _ _ - _ _ _ _ | O no<br>O yes |
| 19. | ……………… | ………………… | O physician<br>O DM | ………………… | _ _ - _ _ - _ _ _ _ | _ _ - _ _ - _ _ _ _ | O no<br>O yes |
| 20. | ……………… | ………………… | O physician<br>O DM | ………………… | _ _ - _ _ - _ _ _ _ | _ _ - _ _ - _ _ _ _ | O no<br>O yes |
| 21. | ……………… | ………………… | O physician<br>O DM | ………………… | _ _ - _ _ - _ _ _ _ | _ _ - _ _ - _ _ _ _ | O no<br>O yes |
| 22. | ……………… | ………………… | O physician<br>O DM | ………………… | _ _ - _ _ - _ _ _ _ | _ _ - _ _ - _ _ _ _ | O no<br>O yes |
| 23. | ……………… | ………………… | O physician<br>O DM | ………………… | _ _ - _ _ - _ _ _ _ | _ _ - _ _ - _ _ _ _ | O no<br>O yes |
| 24. | ……………… | ………………… | O physician<br>O DM | ………………… | _ _ - _ _ - _ _ _ _ | _ _ - _ _ - _ _ _ _ | O no<br>O yes |
| 25. | ……………… | ………………… | O physician<br>O DM | ………………… | _ _ - _ _ - _ _ _ _ | _ _ - _ _ - _ _ _ _ | O no<br>O yes |

| | Toxicity<br>If not specified by the physi-cian, consult terminology used in the PDF file mentioned above | CTC grade<br>If not specified by the physician, consult the guide-lines in the PDF file mentioned above to find the specific CTC grade for the specific toxicity | Determined by | Treatment to which the toxicity is related* | Start date of toxicity | End date of toxicity | Is toxicity treated? |
|---|---|---|---|---|---|---|---|
| 26. | .................. | ..................... | O physician<br>O DM | ..................... | _ _ - _ _ - _ _ _ _ | _ _ - _ _ - _ _ _ _ | O no<br>O yes |
| 27. | .................. | ..................... | O physician<br>O DM | ..................... | _ _ - _ _ - _ _ _ _ | _ _ - _ _ - _ _ _ _ | O no<br>O yes |
| 28. | .................. | ..................... | O physician<br>O DM | ..................... | _ _ - _ _ - _ _ _ _ | _ _ - _ _ - _ _ _ _ | O no<br>O yes |
| 29. | .................. | ..................... | O physician<br>O DM | ..................... | _ _ - _ _ - _ _ _ _ | _ _ - _ _ - _ _ _ _ | O no<br>O yes |
| 30. | .................. | ..................... | O physician<br>O DM | ..................... | _ _ - _ _ - _ _ _ _ | _ _ - _ _ - _ _ _ _ | O no<br>O yes |
| 31. | .................. | ..................... | O physician<br>O DM | ..................... | _ _ - _ _ - _ _ _ _ | _ _ - _ _ - _ _ _ _ | O no<br>O yes |

*** Related to:**
01 = Interferon-alpha (IFN-a)
02 = IFN-a + vinblastin
03 = Interleukin 2
04 = Sunitinib
05 = Sorafenib
06 = Bevacizumab + IFN-a
07 = Temsirolimus
08 = Everolimus
09 = Pazopanib
10 = Radiotherapy
11 = Surgery
88 = other, please specify below
99 = unknown

Related to, option 88, specification:

..........................................................................................................................................

..........................................................................................................................................

..........................................................................................................................................

## Response (also in case of non treatment)

Register the response as mentioned in the RADIOLOGY report; as a substitute use the response as indicated by the physician in the medical record.
Information on response is required, and therefore, this part should always be filled out. Details of tumor assessments (in the part 'tumor assessment': 'date tumor assessment', 'target lesions' and 'non-target lesions' below) should be registered if available.
More information on RECIST version 1.1: www.eortc.be/recist/

| | **Date of response assessment** | **Treatment line** | **Response*** | **In case of PR or SD: Decrease according to RECIST** | **Date of relapse or progression** | **Further anti cancer treatment** | **Reason no further treatment**** |
|---|---|---|---|---|---|---|---|
| | Date on which the response has been established, within 3 months of treatment. This is the date that the physician recorded the response in the medical record. If this is not listed, then record the date of the investigations that are required for the determination of response. Please note the last date; therefore, the date on which all of the required results were available. | 1 = 1st line<br>2 = 2nd line<br>3 = 3rd line<br>4 = 4th line<br>5 = 5th line | 0 = no (drug) treatment<br>1 = CR complete remission<br>2 = PR partial remission<br>3 = SD stable disease<br>4 = PD progressive disease<br>9 = unknown | | Date on which a possible relapse or progress occurs, mostly based on radiology. If no relapse or progress has occured at end of follow-up, fill in 00-00-0000 (not applicable). | Indicate whether the patient receives active treatment as a result of relapse or progression of disease (or when a previous treatment was ineffective). 0= **no** (in case of best supportive care). | If a patient does not receive active treatment (anymore), register the reason why. Multiple reasons are possible. If the field code 88 is filled in, a specification must be given in the available field. |
| 1. | _ _ - _ _ - _ _ _ _ | ............... | ............... | ...............% | _ _ - _ _ - _ _ _ _ | O no<br>O yes | ............... |
| 2. | _ _ - _ _ - _ _ _ _ | ............... | ............... | ...............% | _ _ - _ _ - _ _ _ _ | O no<br>O yes | ............... |
| 3. | _ _ - _ _ - _ _ _ _ | ............... | ............... | ...............% | _ _ - _ _ - _ _ _ _ | O no<br>O yes | ............... |
| 4. | _ _ - _ _ - _ _ _ _ | ............... | ............... | ...............% | _ _ - _ _ - _ _ _ _ | O no<br>O yes | ............... |
| 5. | _ _ - _ _ - _ _ _ _ | ............... | ............... | ...............% | _ _ - _ _ - _ _ _ _ | O no<br>O yes | ............... |
| 6. | _ _ - _ _ - _ _ _ _ | ............... | ............... | ...............% | _ _ - _ _ - _ _ _ _ | O no<br>O yes | ............... |
| 7. | _ _ - _ _ - _ _ _ _ | ............... | ............... | ...............% | _ _ - _ _ - _ _ _ _ | O no<br>O yes | ............... |
| 8. | _ _ - _ _ - _ _ _ _ | ............... | ............... | ...............% | _ _ - _ _ - _ _ _ _ | O no<br>O yes | ............... |
| 9. | _ _ - _ _ - _ _ _ _ | ............... | ............... | ...............% | _ _ - _ _ - _ _ _ _ | O no<br>O yes | ............... |
| 10. | _ _ - _ _ - _ _ _ _ | ............... | ............... | ...............% | _ _ - _ _ - _ _ _ _ | O no<br>O yes | ............... |
| 11. | _ _ - _ _ - _ _ _ _ | ............... | ............... | ...............% | _ _ - _ _ - _ _ _ _ | O no<br>O yes | ............... |

| | Date of response assessment | Treatment line | Response* | In case of PR or SD: Decrease according to RECIST | Date of relapse or progression | Further anti cancer treatment | Reason no further treatment** |
|---|---|---|---|---|---|---|---|
| 12. | _ _ - _ _ - _ _ _ _ | …………… | …………… | ……………% | _ _ - _ _ - _ _ _ _ | O no<br>O yes | …………… |
| 13. | _ _ - _ _ - _ _ _ _ | …………… | …………… | ……………% | _ _ - _ _ - _ _ _ _ | O no<br>O yes | …………… |
| 14. | _ _ - _ _ - _ _ _ _ | …………… | …………… | ……………% | _ _ - _ _ - _ _ _ _ | O no<br>O yes | …………… |
| 15. | _ _ - _ _ - _ _ _ _ | …………… | …………… | ……………% | _ _ - _ _ - _ _ _ _ | O no<br>O yes | …………… |
| 16. | _ _ - _ _ - _ _ _ _ | …………… | …………… | ……………% | _ _ - _ _ - _ _ _ _ | O no<br>O yes | …………… |

*** Response**

0 = No (drug) treatment, adjuvant treatment or palliative treatment whereby the patient has died before the first evaluation time, therefore not applicable
1 = Complete remission (CR): Disappearance of all (target and non-target) lesions
2 = Partial remission (PR): At least 30% decrease in longest diameter (LD) of single tumor or sum of LDs of multiple masses (specify the decrease according to RECIST version 1.1)
3 = Stable disease (SD): Neither PR nor PD criteria met (specify the decrease (if any) according to RECIST version 1.1)
4 = Progressive disease (PD): Greater than 20% increase in LD of single tumor or the sum of LDs in multiple masses, or appearance of new lesions
9 = unknown (also when treatment was not completed)

**** Reason no further treatment**

00 = new treatment starts, so n.a.
01 = no further treatment possibilities
02 = wait-and-see policy
03 = refusal patient / family
04 = co morbidity
05 = functional status
06 = age
07 = short life expectancy
08 = patient has died
88 = other, please specify below
99 = unknown

Reason no further treatment, option 88, specification:

……………………………………………………………………………………………………………………………

……………………………………………………………………………………………………………………………

……………………………………………………………………………………………………………………………

## Tumor assessment (also in case of non treatment)

Details on ‘tumor assessment’, in the parts ‘date tumor assessment’, ‘target lesions’ and ‘non-target lesions’ should be filled out, if available. So please fill out the dates on which tumor assessments were performed in this treatment line and provide detailed information on target and non-target lesions.
Click on the + buttons to add a new assessment.

## Date tumor assessment

| | | Date |
|---|---|---|
| 1. | Baseline assessment | __ __ -- __ __ -- __ __ __ __ |
| 2. | Tumor assessment 2 | __ __ -- __ __ -- __ __ __ __ |
| 3. | Tumor assessment 3 | __ __ -- __ __ -- __ __ __ __ |
| 4. | Tumor assessment 4 | __ __ -- __ __ -- __ __ __ __ |
| 5. | Tumor assessment 5 | __ __ -- __ __ -- __ __ __ __ |
| 6. | Tumor assessment 6 | __ __ -- __ __ -- __ __ __ __ |
| 7. | Tumor assessment 7 | __ __ -- __ __ -- __ __ __ __ |
| 8. | Tumor assessment 8 | __ __ -- __ __ -- __ __ __ __ |
| 9. | Tumor assessment 9 | __ __ -- __ __ -- __ __ __ __ |
| 10. | Tumor assessment 10 | __ __ -- __ __ -- __ __ __ __ |
| 11. | Tumor assessment 11 | __ __ -- __ __ -- __ __ __ __ |
| 12. | Tumor assessment 12 | __ __ -- __ __ -- __ __ __ __ |
| 13. | Tumor assessment 13 | __ __ -- __ __ -- __ __ __ __ |
| 14. | Tumor assessment 14 | __ __ -- __ __ -- __ __ __ __ |
| 15. | Tumor assessment 15 | __ __ -- __ __ -- __ __ __ __ |
| 16. | Tumor assessment 16 | __ __ -- __ __ -- __ __ __ __ |

## Target Lesions

All measurable lesions up to a maximum of 5 lesions (2 per organ) representative of all involved organs should be identified as target lesions and be recorded and measured at baseline. These 5 lesions should be selected on the basis of their size (lesions with the longest diameter) and their suitability for accurate repetitive measurements (either by imaging techniques or clinically). A sum of the longest diameter (LD) for all target lesions will be calculated and reported as the baseline sum LD. The baseline sum LD will be used as reference to further characterize the objective tumor response of the measurable dimension of the disease. If there are >5 measurable lesions, those not selected as target lesions will be considered together with non-measurable disease as non-target lesions.

Kidney-ipsilateral = side of primary tumor
For lymph nodes: Take shortest diameter

| | Location /type | Target lesion 1 | Target lesion 2 | Target lesion 3 | Target lesion 4 | Target lesion 5 | Sum of lesion diameters | Overall Response |
|---|---|---|---|---|---|---|---|---|
| 1 | Location /type<br>ipsi = ipsilateral<br>con = contra-lateral<br>adrenal g = adrenal gland | Okidney ipsi<br>Okidney con<br>Olung<br>Olymphnode<br>Oliver<br>Obrain<br>Oadrenal g<br>Oother*<br>On.a. | Okidney ipsi<br>Okidney con<br>Olung<br>Olymphnode<br>Oliver<br>Obrain<br>Oadrenal g<br>Oother*<br>On.a. | Okidney ipsi<br>Okidney con<br>Olung<br>Olymphnode<br>Oliver<br>Obrain<br>Oadrenal g<br>Oother*<br>On.a. | Okidney ipsi<br>Okidney con<br>Olung<br>Olymphnode<br>Oliver<br>Obrain<br>Oadrenal g<br>Oother*<br>On.a. | Okidney ipsi<br>Okidney con<br>Olung<br>Olymphnode<br>Oliver<br>Obrain<br>Oadrenal g<br>Oother*<br>On.a. | | CR (complete remission)<br>PR (partial remission)<br>SD (stable disease)<br>PD (pro-gressive disease)<br>U (unknown) |
| 2 | Baseline assessment | ……… mm | ……… mm | ……… mm | ……… mm | ……… mm | …… mm | O CR O PD<br>O PR O U<br>O SD |
| 3 | Tumor 2 assessment | ……… mm | ……… mm | ……… mm | ……… mm | ……… mm | …… mm | O CR O PD<br>O PR O U<br>O SD |
| 4 | Tumor 3 assessment | ……… mm | ……… mm | ……… mm | ……… mm | ……… mm | …… mm | O CR O PD<br>O PR O U<br>O SD |
| 5 | Tumor 4 assessment | ……… mm | ……… mm | ……… mm | ……… mm | ……… mm | …… mm | O CR O PD<br>O PR O U<br>O SD |
| 6 | Tumor 5 assessment | ……… mm | ……… mm | ……… mm | ……… mm | ……… mm | …… mm | O CR O PD<br>O PR O U<br>O SD |
| 7 | Tumor 6 assessment | ……… mm | ……… mm | ……… mm | ……… mm | ……… mm | …… mm | O CR O PD<br>O PR O U<br>O SD |
| 8 | Tumor 7 assessment | ……… mm | ……… mm | ……… mm | ……… mm | ……… mm | …… mm | O CR O PD<br>O PR O U<br>O SD |
| 9 | Tumor 8 assessment | ……… mm | ……… mm | ……… mm | ……… mm | ……… mm | …… mm | O CR O PD<br>O PR O U<br>O SD |
| 10 | Tumor 9 assessment | ……… mm | ……… mm | ……… mm | ……… mm | ……… mm | …… mm | O CR O PD<br>O PR O U<br>O SD |
| 11 | Tumor 10 assessment | ……… mm | ……… mm | ……… mm | ……… mm | ……… mm | …… mm | O CR O PD<br>O PR O U<br>O SD |

| | | | | | | | | |
|---|---|---|---|---|---|---|---|---|
| 12 | Tumor 11 assessment | ……… mm | ……… mm | ……… mm | ……… mm | ……… mm | …… mm | O CR O PD O PR O U O SD |
| 13 | Tumor 12 assessment | ……… mm | ……… mm | ……… mm | ……… mm | ……… mm | …… mm | O CR O PD O PR O U O SD |
| 14 | Tumor 13 assessment | ……… mm | ……… mm | ……… mm | ……… mm | ……… mm | …… mm | O CR O PD O PR O U O SD |
| 15 | Tumor 14 assessment | ……… mm | ……… mm | ……… mm | ……… mm | ……… mm | …… mm | O CR O PD O PR O U O SD |
| 16 | Tumor 15 assessment | ……… mm | ……… mm | ……… mm | ……… mm | ……… mm | …… mm | O CR O PD O PR O U O SD |
| 17 | Tumor 16 assessment | ……… mm | ……… mm | ……… mm | ……… mm | ……… mm | …… mm | O CR O PD O PR O U O SD |
| *Target lesion other | | …………… | …………… | …………… | …………… | …………… | | |

## Non-Target Lesions

All non-measurable lesions (or sites of disease) plus any measurable lesions over and above the 5 listed as target lesions. Measurements are not required but these lesions should be noted at baseline and should be followed as "equal", "increase" or "decrease".

| | **Location /type** | **Non-target lesion 1** | **Non-target lesion 2** | **Non-target lesion 3** | **Non-target lesion 4** | **Non-target lesion 5** |
|---|---|---|---|---|---|---|
| 1 | Location /type<br><br>ipsi = ipsilateral<br>contr = contra-lateral<br>adrenal gl = adrenal gland | O kidney ipsi<br>O kidney contr<br>O lung<br>O lymphnodes<br>O liver<br>O bone<br>O brain<br>O adrenal gl<br>O other*<br>O n.a. | O kidney ipsi<br>O kidney contr<br>O lung<br>O lymphnodes<br>O liver<br>O bone<br>O brain<br>O adrenal gl<br>O other*<br>O n.a. | O kidney ipsi<br>O kidney contr<br>O lung<br>O lymphnodes<br>O liver<br>O bone<br>O brain<br>O adrenal gl<br>O other*<br>O n.a. | O kidney ipsi<br>O kidney contr<br>O lung<br>O lymphnodes<br>O liver<br>O bone<br>O brain<br>O adrenal gl<br>O other*<br>O n.a. | O kidney ipsi<br>O kidney contr<br>O lung<br>O lymphnodes<br>O liver<br>O bone<br>O brain<br>O adrenal gl<br>O other*<br>O n.a. |
| 2 | Baseline assessment | O no<br>O yes | O no<br>O yes | O no<br>O yes | O no<br>O yes | O no<br>O yes |
| 3 | Tumor 2 assessment | O equal<br>O increase<br>O decrease | O equal<br>O increase<br>O decrease | O equal<br>O increase<br>O decrease | O equal<br>O increase<br>O decrease | O equal<br>O increase<br>O decrease |
| 4 | Tumor 3 assessment | O equal<br>O increase<br>O decrease | O equal<br>O increase<br>O decrease | O equal<br>O increase<br>O decrease | O equal<br>O increase<br>O decrease | O equal<br>O increase<br>O decrease |
| 5 | Tumor 4 assessment | O equal<br>O increase<br>O decrease | O equal<br>O increase<br>O decrease | O equal<br>O increase<br>O decrease | O equal<br>O increase<br>O decrease | O equal<br>O increase<br>O decrease |
| 6 | Tumor 5 assessment | O equal<br>O increase<br>O decrease | O equal<br>O increase<br>O decrease | O equal<br>O increase<br>O decrease | O equal<br>O increase<br>O decrease | O equal<br>O increase<br>O decrease |
| 7 | Tumor 6 assessment | O equal<br>O increase<br>O decrease | O equal<br>O increase<br>O decrease | O equal<br>O increase<br>O decrease | O equal<br>O increase<br>O decrease | O equal<br>O increase<br>O decrease |
| 8 | Tumor 7 assessment | O equal<br>O increase<br>O decrease | O equal<br>O increase<br>O decrease | O equal<br>O increase<br>O decrease | O equal<br>O increase<br>O decrease | O equal<br>O increase<br>O decrease |
| 9 | Tumor 8 assessment | O equal<br>O increase<br>O decrease | O equal<br>O increase<br>O decrease | O equal<br>O increase<br>O decrease | O equal<br>O increase<br>O decrease | O equal<br>O increase<br>O decrease |
| 10 | Tumor 9 assessment | O equal<br>O increase<br>O decrease | O equal<br>O increase<br>O decrease | O equal<br>O increase<br>O decrease | O equal<br>O increase<br>O decrease | O equal<br>O increase<br>O decrease |
| 11 | Tumor 10 assessment | O equal<br>O increase<br>O decrease | O equal<br>O increase<br>O decrease | O equal<br>O increase<br>O decrease | O equal<br>O increase<br>O decrease | O equal<br>O increase<br>O decrease |
| 12 | Tumor 11 assessment | O equal<br>O increase<br>O decrease | O equal<br>O increase<br>O decrease | O equal<br>O increase<br>O decrease | O equal<br>O increase<br>O decrease | O equal<br>O increase<br>O decrease |

| | Location /type | Non-target lesion 1 | Non-target lesion 2 | Non-target lesion 3 | Non-target lesion 4 | Non-target lesion 5 |
|---|---|---|---|---|---|---|
| 13 | Tumor 12 assessment | O equal<br>O increase<br>O decrease | O equal<br>O increase<br>O decrease | O equal<br>O increase<br>O decrease | O equal<br>O increase<br>O decrease | O equal<br>O increase<br>O decrease |
| 14 | Tumor 13 assessment | O equal<br>O increase<br>O decrease | O equal<br>O increase<br>O decrease | O equal<br>O increase<br>O decrease | O equal<br>O increase<br>O decrease | O equal<br>O increase<br>O decrease |
| 15 | Tumor 14 assessment | O equal<br>O increase<br>O decrease | O equal<br>O increase<br>O decrease | O equal<br>O increase<br>O decrease | O equal<br>O increase<br>O decrease | O equal<br>O increase<br>O decrease |
| 16 | Tumor 15 assessment | O equal<br>O increase<br>O decrease | O equal<br>O increase<br>O decrease | O equal<br>O increase<br>O decrease | O equal<br>O increase<br>O decrease | O equal<br>O increase<br>O decrease |
| 17 | Tumor 16 assessment | O equal<br>O increase<br>O decrease | O equal<br>O increase<br>O decrease | O equal<br>O increase<br>O decrease | O equal<br>O increase<br>O decrease | O equal<br>O increase<br>O decrease |
| *Target lesion other | | ……………… | ……………… | ……………… | ……………… | ……………… |

## New Lesions

| | |
|---|---|
| New lesion during this treatment line? | O no<br>O yes |
| Date new lesion | __ __ -- __ __ -- __ __ __ __ |

## Final information on patient

Final information on patient

This form should be filled in when a patient has died or is lost to follow up

| | |
|---|---|
| At which date has the final information (toxicities, treatment, etc.) been collected | __ __ -- __ __ -- __ __ __ __ |
| Reason for last registration | O Lost to follow up (e.g., referral to another hospital, registration impossible)<br>O Patient has died<br>O Unknown |
| In case the patient is lost to follow up, last date patient was known to be alive | __ __ -- __ __ -- __ __ __ __ |
| In case the patient has died, date of death | __ __ -- __ __ -- __ __ __ __ |
| In case the patient has died, cause of death | O As a result of the malignancy<br>O Due to toxicity (specify in “toxicities and response”)<br>O Other, please specify below<br>O Unknown |
| Other cause of death, option 8 | ……………………………………………………………………<br>……………………………………………………………………<br>…………………………………………………………………… |
| Remarks | ……………………………………………………………………<br>……………………………………………………………………<br>…………………………………………………………………… |
