# Supplementary material for: Genome-Wide Meta-Analysis Identifies Variants in DSCAM and PDLIM3 That Correlate with Efficacy Outcomes in Metastatic Renal Cell Carcinoma Patients Treated with Sunitinib
Source: Cancers (Basel). 2022 Jun 8;14(12):2838. doi: 10.3390/cancers14122838 (PMC9220885; doi:10.3390/cancers14122838)
Supplement: Supplementary file 1 [file cancers-14-02838-s001.zip › Supplementary File S1b.pdf]

Web-based Case Record Forms

EuroTARGET portal

Clinical baseline and follow-up data of EuroTARGET patients will be collected in web-based case record forms (CRFs). These web-based CRFs can be filled out via the user-friendly EuroTARGET portal: **https://eurotarget.glean.nl**

The portal can be entered with your personal account. Please change your password the first time you login. A password has to:

- Be at least 10 characters
- Of which at least 2 numbers
- And at least 2 special characters
- And at least 6 letters, of which at least 1 should be capitalized

You will only have access to patients of your own institute.

Please ignore the Dutch Announcements! Announcements will be communicated to you via e-mail.

Please “**log out**” after you are finished. Just closing the application may give problems the next time you are logging in.

EuroTARGET study number

Once a new patient has been added a EuroTARGET study number will be generated automatically. Please use this EuroTARGET study number on blood and tumor samples.

CRFs

After adding a new patient the following CRFs will become available for this patient:

- **Eligibility criteria:**
  This information should be registered once for each patient at time of recruitment.

- **General information at diagnosis:**
  Information on "tumor assessment" and "co-morbidities at start treatment of metastasis". This information should be registered once for each patient at time of recruitment.

- **Available patient material:**
  Please register whether "blood sample" and "tumor sample" are available for EuroTARGET.

- **Treatment line:**
  Information collected during treatment regimes for metastasis. When another drug is administered a new treatment line starts. Please use one form per treatment line. “1st line treatment of metastasis” should always be filled out. “2nd, 3rd, 4th and 5th line treatment of metastasis” forms are exact copies of “1st line treatment of metastasis” and should only be filled out in case of (multiple) medication change(s). Information

on "toxicities" and "response" must be updated on an ongoing basis (at least once every 6 months), preferably after each check-up.
In the table "response (also in case of non treatment)", the column 'In case of PR or SD: Decrease according to RECIST' does NOT need to be filled out. In addition, the parts "Tumor assessment (also in case of non treatment)", "Date tumor assessment", "Target lesions", and "Non-target lesions" do NOT need to be filled out.

- **Questionnaire data:**
  Please insert data from the patient questionnaire. Information on "smoking habits", "family history", "medication", all prior to study entry. In case NO questionnaire is available (e.g., in case of retrospective data), just leave the status of the CRF at "unstarted".
- **Final information on patient:**
  This information should be registered when a patient has died or is lost to follow up.

CRFs – finished

CRFs that are not started yet will have the status "unstarted". After entering and saving data the status of the CRF will become "unfinished". You can save a partially filled out CRF and complete it later.

The CRF "treatment line" needs to be updated during follow up of the patient. Follow up ends when a patient dies, is lost to follow up, or at the end of the study. When follow-up is not complete yet, leave the status of the CRF at "unfinished".

After all fields have been completed please put the status of the CRF on "finished" (at right top corner of the CRF). The system will then check whether all fields are filled in correctly and "sentdate" will be generated automatically.

Export function

The homepage (begin screen) of the EuroTARGET portal has the option "Export eCRF". To export data of your patients, click this button, leave all export format options in the default settings, and click "export". This will generate a folder with 2 Excel files per CRF; one with the data, and one with information about the variables. To export not only the CRFs, but also the Patient Data, you will need to check that option in the export format options.

Test account

You can take a look at the CRFs of a test patient by logging in on the portal with the test account:

Username: et_testphys
Password: Testphys11!!

Please do NOT change the password and do NOT enter real patients for this test institute!

Please "**log out**" after you are finished. Just closing the application may give problems the next time you or another test account user are logging in.

Questions

For questions please contact Dr Loes van der Zanden at Loes.vanderZanden@radboudumc.nl
