# Supplementary material for: Genome-Wide Meta-Analysis Identifies Variants in DSCAM and PDLIM3 That Correlate with Efficacy Outcomes in Metastatic Renal Cell Carcinoma Patients Treated with Sunitinib
Source: Cancers (Basel). 2022 Jun 8;14(12):2838. doi: 10.3390/cancers14122838 (PMC9220885; doi:10.3390/cancers14122838)
Supplement: Supplementary file 1 [file cancers-14-02838-s001.zip › Supplementary File S2.pdf]

Supplement 2: Linkage Disequilibrium (LD) with R^2 values for *DSCAM* gene variants.

Supplement 2a: Overview of genetic variants in DSCAM that are in linkage disequilibrium with a R^2>0.8. The values were obtained using the LD proxy tool from the LDlink website of the National Cancer Institute (https://ldlink.nci.nih.gov/?tab=ldproxy), in which SNP rs2205096 in *DSCAM* in the European population was entered.

RS number	Coord	Alleles	MAF	Distance	Dprime	R2	Correlated_Alleles	RegulomeDB
rs2205096	chr21:41683405	(A/G)	0.0646	0	1.0	1.0	A=A,G=G	7
rs8126659	chr21:41682972	(C/T)	0.0636	-433	1.0	0.9836	A=C,G=T	6
rs1554923	chr21:41682817	(C/A)	0.0636	-588	1.0	0.9836	A=C,G=A	7
rs2205095	chr21:41676786	(T/A)	0.0616	-6619	0.9828	0.9183	A=T,G=A	6
rs2837569	chr21:41680306	(G/T)	0.0596	-3099	1.0	0.9182	A=G,G=T	5
rs1159719	chr21:41670971	(C/T)	0.0626	-12434	0.9661	0.9026	A=C,G=T	6
rs61349484	chr21:41668658	(G/T)	0.0626	-14747	0.9661	0.9026	A=G,G=T	5
rs73227032	chr21:41673539	(A/G)	0.0606	-9866	0.9649	0.8701	A=A,G=G	5
rs68151839	chr21:41673323	(A/G)	0.0606	-10082	0.9649	0.8701	A=A,G=G	7
rs66765790	chr21:41673200	(G/C)	0.0606	-10205	0.9649	0.8701	A=G,G=C	7
rs2837565	chr21:41672532	(T/C)	0.0606	-10873	0.9649	0.8701	A=T,G=C	4
rs2837564	chr21:41672173	(T/C)	0.0606	-11232	0.9649	0.8701	A=T,G=C	6
rs2837563	chr21:41671977	(T/A)	0.0606	-11428	0.9649	0.8701	A=T,G=A	6
rs2837562	chr21:41671906	(C/G)	0.0606	-11499	0.9649	0.8701	A=C,G=G	7
rs2837561	chr21:41671704	(A/T)	0.0606	-11701	0.9649	0.8701	A=A,G=T	6
rs7282745	chr21:41671653	(T/G)	0.0606	-11752	0.9649	0.8701	A=T,G=G	6
rs2837560	chr21:41671275	(G/A)	0.0606	-12130	0.9649	0.8701	A=G,G=A	7
rs2837559	chr21:41671015	(C/T)	0.0606	-12390	0.9649	0.8701	A=C,G=T	7
rs2837558	chr21:41670535	(T/C)	0.0606	-12870	0.9649	0.8701	A=T,G=C	7
rs2837557	chr21:41670497	(A/G)	0.0606	-12908	0.9649	0.8701	A=A,G=G	6
rs2837556	chr21:41670440	(G/C)	0.0606	-12965	0.9649	0.8701	A=G,G=C	7
rs7276530	chr21:41670422	(G/A)	0.0606	-12983	0.9649	0.8701	A=G,G=A	7
rs7276117	chr21:41670303	(C/T)	0.0606	-13102	0.9649	0.8701	A=C,G=T	6
rs7277178	chr21:41670289	(T/G)	0.0606	-13116	0.9649	0.8701	A=T,G=G	7
rs7282179	chr21:41670052	(T/C)	0.0606	-13353	0.9649	0.8701	A=T,G=C	7
rs7278717	chr21:41674286	(G/C)	0.0616	-9119	0.9483	0.855	A=G,G=C	6
rs714983	chr21:41674094	(T/G)	0.0616	-9311	0.9483	0.855	A=T,G=G	5
rs1537110	chr21:41672463	(C/G)	0.0616	-10942	0.9483	0.855	A=C,G=G	4
rs8128963	chr21:41672394	(G/A)	0.0616	-11011	0.9483	0.855	A=G,G=A	4

rs2837566	chr21:41673535	(G/A)	0.0626	-9870	0.9321	0.8403	A=G,G=A	5
rs714982	chr21:41674108	(T/G)	0.0606	-9297	0.9474	0.8388	A=T,G=G	5
rs150942709	chr21:41678694	(A/C)	0.0765	-4711	1.0	0.8334	A=A,G=C	7
rs57108956	chr21:41674465	(A/G)	0.0765	-8940	1.0	0.8334	A=A,G=G	5

Supplement 2b: Overview of genetic variants in DSCAM that are in linkage disequilibrium with a R^2>0.8. The values were obtained using HaploReg version 4.1 (http://pubs.broadinstitute.org/mammals/haploreg/haploreg.php) from Massachusetts Institute of Technology (MIT), in which SNP rs2205096 in *DSCAM* in the European population was entered.

chr	pos (hg38)	LD (r^2)	LD (D')	variant	Ref	Alt	AFR freq	AMR freq	ASN freq	EUR freq	DNAse	Proteins bound	Motifs changed	GENCODE genes	dbSNP func annot
21	40296731	0.82	0.93	rs61349484	G	T	0.57	0.16	0.34	0.06			6 altered motifs	DSCAM	intronic
21	40298125	0.84	0.97	rs7282179	T	C	0.45	0.15	0.37	0.05			Lmo2-complex,RFX5,TCF12	DSCAM	intronic
21	40298376	0.84	0.97	rs7276117	C	T	0.45	0.15	0.37	0.05			Mtf1,Myc,p53	DSCAM	intronic
21	40298495	0.84	0.97	rs7276530	G	A	0.45	0.15	0.37	0.05			Pou1f1	DSCAM	intronic
21	40298513	0.84	0.97	rs2837556	G	C	0.45	0.15	0.37	0.05			CEBPG	DSCAM	intronic
21	40298570	0.84	0.97	rs2837557	A	G	0.45	0.15	0.37	0.05			AP-1,CACD,CEBPD	DSCAM	intronic
21	40298608	0.84	0.97	rs2837558	T	C	0.45	0.15	0.37	0.05			Foxj1,Otx2	DSCAM	intronic
21	40299044	0.86	0.97	rs1159719	C	T	0.49	0.16	0.37	0.05			Ik-1,NF-kappaB,RBP-Jkappa	DSCAM	intronic
21	40299088	0.84	0.97	rs2837559	C	T	0.45	0.15	0.37	0.05			Gfi1,Gfi1b	DSCAM	intronic
21	40299348	0.84	0.97	rs2837560	G	A	0.46	0.15	0.37	0.05			7 altered motifs	DSCAM	intronic
21	40299726	0.84	0.97	rs7282745	T	G	0.46	0.15	0.37	0.05	MUS		4 altered motifs	DSCAM	intronic
21	40299777	0.84	0.97	rs2837561	A	T	0.45	0.15	0.37	0.05	MUS		TCF4	DSCAM	intronic
21	40299979	0.84	0.97	rs2837562	C	G	0.46	0.15	0.37	0.05	MUS		Nkx2	DSCAM	intronic
21	40300050	0.84	0.97	rs2837563	T	A	0.45	0.15	0.37	0.05			CEBPG	DSCAM	intronic
21	40300246	0.84	0.97	rs2837564	T	C	0.43	0.15	0.37	0.05			GCNF,Nr2e3,YY1	DSCAM	intronic
21	40300467	0.84	0.97	rs8128963	G	A	0.46	0.15	0.37	0.05	ESDR,ESDR,HRT	NRSF	HES1	DSCAM	intronic
21	40300536	0.84	0.97	rs1537110	C	G	0.46	0.15	0.37	0.05	ESDR,ESC	NRSF	CCNT2,MAZR	DSCAM	intronic

21	40300605	0.84	0.97	rs2837565	T	C	0.46	0.15	0.37	0.05		NRSF,CTCF	4 altered motifs	DSCAM	intronic
21	40301273	0.84	0.97	rs66765790	G	C	0.46	0.15	0.37	0.05			Pou2f2,Zfp410	DSCAM	intronic
21	40301396	0.84	0.97	rs68151839	A	G	0.46	0.15	0.37	0.05			BDP1	DSCAM	intronic
21	40301753	0.81	0.97	rs67162236	C	T	0.16	0.07	0.13	0.05	SKIN		DMRT1	DSCAM	intronic
21	40302167	0.93	0.98	rs714983	T	G	0.34	0.14	0.37	0.06			7 altered motifs	DSCAM	intronic
21	40302181	0.93	0.98	rs714982	T	G	0.34	0.14	0.37	0.06				DSCAM	intronic
21	40302359	0.93	0.98	rs7278717	G	C	0.34	0.14	0.37	0.06	SKIN		BCL,NF-kappaB	DSCAM	intronic
21	40302887	0.86	0.97	rs2837567	C	T	0.31	0.13	0.37	0.05			13 altered motifs	DSCAM	intronic
21	40304859	0.82	0.93	rs2205095	T	A	0.41	0.15	0.37	0.06			Dlx2,Osr,Sox	DSCAM	intronic
21	40307547	0.82	0.98	rs4411799	T	C	0.54	0.16	0.37	0.07			Ets,Zbtb12	DSCAM	intronic
21	40307920	0.81	1	rs113168647	C	T	0.04	0.07	0.12	0.05			EBF,Myb	DSCAM	intronic
21	40308379	0.88	1	rs2837569	G	T	0.51	0.17	0.37	0.05	HRT		Egr-1,Gfi1,Hsf	DSCAM	intronic
21	40310890	0.98	1	rs1554923	C	A	0.52	0.17	0.37	0.06				DSCAM	intronic
21	40311045	0.98	1	rs8126659	C	T	0.51	0.17	0.36	0.06			LBP-1,Nanog,TCF12	DSCAM	intronic
21	40311478	1	1	rs2205096	A	G	0.51	0.17	0.37	0.06				DSCAM	intronic
